# Complex Interplay of Heme-Copper Oxidases with Nitrite and Nitric Oxide

**DOI:** 10.3390/ijms23020979

**Published:** 2022-01-17

**Authors:** Jinghua Chen, Peilu Xie, Yujia Huang, Haichun Gao

**Affiliations:** Institute of Microbiology, College of Life Sciences, Zhejiang University, Hangzhou 310058, China; cjh188@zju.edu.cn (J.C.); 21907023@zju.edu.cn (P.X.); 3180102131@zju.edu.cn (Y.H.)

**Keywords:** nitrite, nitric oxide, heme-copper oxidase, proton-pumping

## Abstract

Nitrite and nitric oxide (NO), two active and critical nitrogen oxides linking nitrate to dinitrogen gas in the broad nitrogen biogeochemical cycle, are capable of interacting with redox-sensitive proteins. The interactions of both with heme-copper oxidases (HCOs) serve as the foundation not only for the enzymatic interconversion of nitrogen oxides but also for the inhibitory activity. From extensive studies, we now know that NO interacts with HCOs in a rapid and reversible manner, either competing with oxygen or not. During interconversion, a partially reduced heme/copper center reduces the nitrite ion, producing NO with the heme serving as the reductant and the cupric ion providing a Lewis acid interaction with nitrite. The interaction may lead to the formation of either a relatively stable nitrosyl-derivative of the enzyme reduced or a more labile nitrite-derivative of the enzyme oxidized through two different pathways, resulting in enzyme inhibition. Although nitrite and NO show similar biochemical properties, a growing body of evidence suggests that they are largely treated as distinct molecules by bacterial cells. NO seemingly interacts with all hemoproteins indiscriminately, whereas nitrite shows high specificity to HCOs. Moreover, as biologically active molecules and signal molecules, nitrite and NO directly affect the activity of different enzymes and are perceived by completely different sensing systems, respectively, through which they are linked to different biological processes. Further attempts to reconcile this apparent contradiction could open up possible avenues for the application of these nitrogen oxides in a variety of fields, the pharmaceutical industry in particular.

## 1. Introduction

Proton motive force (pmf) is essential for bacteria to grow and survive under non-replicating conditions by providing energy for a wide range of crucial processes [1,2,3]. The pmf (electrochemical potential) consists of two gradients: the chemical proton or pH gradient (∆pH) and the membrane potential generated by the transport of electrical charge (∆ψ). Bacteria are capable of generating the pmf by a variety of mechanisms; among them, the most efficient one is through oxygen reduction [3]. The oxygen-reducing enzymes (terminal oxidases) that contribute to the pmf generation are classified into two main groups: heme-copper oxidase (HCO) (also called heme-copper oxygen reductase (HCOR)) superfamily and *bd*-type quinol oxidase (*bd* QO) family [4,5].

The HCO superfamily is composed of three subfamilies, A, B, and C, as well as the structurally-related nitric oxide (NO) reductases (NOR) [6,7]. A-family HCOs include cytochrome *c* oxidases (C*c*Os), such as *aa*_3_ from eukaryotic mitochondria, and some prokaryotes (often as *caa*_3_, where *c* represents a cytochrome *c* subunit), and QOs, such as *bo*_3_ of *Escherichia coli* [8,9,10]. HCOs of B and C families are present only in prokaryotes, with *ba*_3_ and *cbb*_3_ as respective representatives [9,11]. HCOs are highly efficient and specialized in pmf generation during the exothermic four-electron reduction of O_2_ because of the proton-pumping mechanism [12,13]. In contrast, *bd* QOs, found exclusively in prokaryotes to date, do not pump protons and are thus less efficient in energy conservation, but play an important role in mediating viability under various stress conditions [14,15].

Although HCOs of A, B, and C families are diverse in terms of subunit composition, electron donor, and heme type, they house a similar signature active site, the so-called binuclear center (BNC), where the reduction chemistry occurs [7]. Located in a subunit with 12 membrane-spanning helices, this BNC consists of two magnetically coupled redox-active metal centers, a high-spin heme (*a*_3_, *o*_3_, or *b*_3_), and a copper ion (Cu_B_) [7,8,11,16]. In all HCOs, these two metal centers are in proximity, with the two metals (Fe and Cu) only ~5 Å apart [7,17]. During the oxygen reduction, the BNC experiences an oxidative-to-reductive phase transition involving several intermediate states [18].

**Figure 1:**
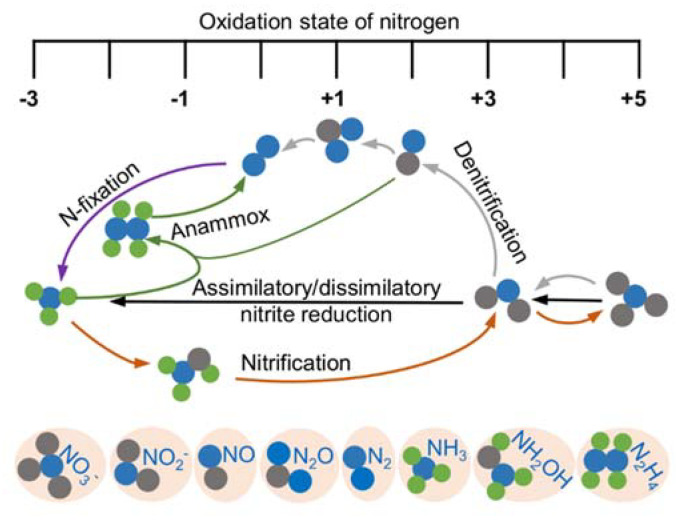
Redox cycle for nitrogen driven by prokaryotes. Shown are the major biological nitrogen transformation pathways, each of which are represented by lines in the same color, and the relative oxidation state at which they occur.

Nitrogen is essential to all life and is a constituent element of amino acids, proteins, and nucleic acids. After fixation, nitrogen as nitrogen gas (N_2_), the most abundant element in the atmosphere, can be converted to ammonium and a variety of nitrogen oxides, among which nitrite (NO_2_^−^) and nitric oxide (NO) are the most common and bioactive species [19,20] (Figure 1). Given that bacteria are able to catalyze all steps of the nitrogen cycle, they are crucial for the inter-conversion of different nitrogen oxides [21]. Both nitrite and NO are involved in diverse physiological processes in bacteria functioning as important cellular signaling molecules, substrates of metabolic enzymes, and inhibitory agents modulating protein activity [19,22]. Although a significant portion of phenotypic changes caused by nitrite are NO-independent, it is widely accepted that NO is the molecule largely underpinning the physiological influences; nitrite impacts the physiology of living organisms in part by serving as a biochemical circulating reservoir for NO [19]. Additionally, NO can be engaged in cellular physiological and pathological processes through a complex cross-talking with two other gasotransmitters, carbon monoxide (CO) and hydrogen sulfide (H_2_S) [23,24,25,26,27]. Meanwhile, nitrite can be converted back to NO through the one-electron-oxidation of NO.

Given the particular importance of the inter-conversion of nitrite and NO, bacteria have evolved a variety of enzymes to catalyze the transformation of nitrite and NO, including NOR [19,28]. In addition, although the HCOs of all A, B, and C families differ from NOR in their metal ions within the BNCs, they are also profoundly implicated in the biology of nitrogen oxides. The direct reactions of HCOs with nitrite and NO, which have been known for a long time, provide a mechanistic understanding of the interplay between the enzymes and the two nitrogen oxides [29,30]. On one hand, it is well known that eukaryotic C*c*Os mediate the reduction of nitrite to NO under hypoxic conditions [31,32]. On the other hand, bacterial HCOs (*ba*_3_, *caa*_3_, *bo*_3_) of the A and B families are capable of catalyzing the reduction of NO to N_2_O, whereas C-family HCOs (*cbb*_3_) convert nitrite to N_2_O [19,33,34,35]. On the other hand, both nitrite and NO are bacteriostatic agents due to their ability to inhibit proteins, especially hemoproteins [36,37]. A great body of evidence has been accumulated showing that nitrite and NO react with purified HCOs and *bd* QOs in vitro and inhibit cell respiration in vivo [22,30,31,38,39,40,41,42]. While there are common reaction mechanisms involved in the inhibition by nitrite and NO, considerable discrepancies have been observed between their cellular targets identified to date [22]. Thus, the extent of the effective mechanisms elucidated by in vitro analyses is in living cells is still a matter of study.

In this review, we aim to present recent findings in the context of the current understanding of the interactions of HCOs with nitrite and NO. It begins with a concise introduction of structural and functional properties of HCOs and *bd* QOs, which serves as the basis for comprehension of the mechanistic characteristics of the interaction. By comparing nitrite and NO, in terms of their biochemical features and physiological impacts in bacteria, we evaluate the presently accepted “avenues” of nitrite and NO and the discrepancy between in vitro and in vivo analyses. The knowledge summarized here encourages future investigations into new potential pathways, new functions, and new mechanisms regarding how living organisms exploit nitrite and NO while preventing their damages.

## 2. Bacterial Terminal Oxidases for pmf Generation

Proton translocation across the membrane is of crucial importance for sustaining the cellular activity in all organisms. However, due to the polarity characteristics, protons are unable to pass through the phospholipid-bilayer membrane freely by diffusion like small non-polar molecules. Proton pumps are special and efficient hydrogen ion transporters that move protons across the membrane from the low-concentration side to the high-concentration side to form pmf, which is subsequently utilized to drive the production of adenosine triphosphate (ATP), the cell’s chemical energy currency, by ATP synthase [1,2,3].

In aerobic bacteria, transmembrane proton pumping is closely related to the oxidative phosphorylation process, especially with the terminal oxidases in the respiratory chain. The bacterial terminal oxidases, including HCOs of the A, B, and C families and *bd* QOs, catalyze the four-electron reduction of oxygen to water using quinol or cytochrome *c* as the electron donors. The main role of most HCOs in microbial metabolism is to conserve energy [43,44,45], and *bd* QOs are thought to contribute to nitrosative stress tolerance, hydrogen peroxide detoxification, or prevention of H_2_S toxicity, especially in pathogenic bacteria [42,46,47,48,49]. To date, a number of high-resolution structures of terminal oxidases from each group have been reported, which have greatly enhanced our understanding of the exact working mechanism of these enzymes.

### 2.1. HCOs

**Figure 2:**
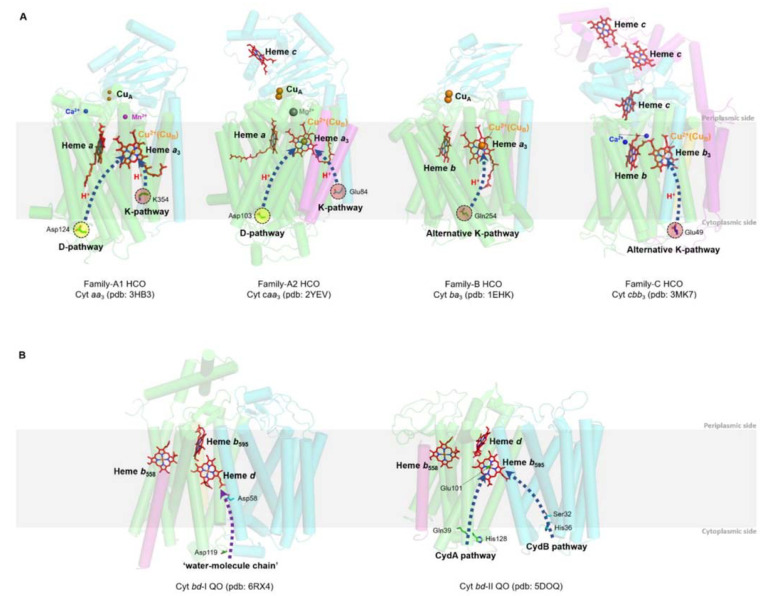
Prosthetic group arrangements and proton pathways of typical bacterial HCOs (**A**) and bd QOs (**B**). (**A**) Representative structures of HCOs of A (divided into A1 and A2), B, and C subfamilies. Protein peptides, heme cofactors, and ions are shown as cartoons, sticks, and spheres, respectively. SUs I of families A and B, SU III of family A and CcoN of family C are colored in green; SUs II of families A and B, and CcoO of family C are colored in cyan; SU IV of family A, SU Iia of family B, and CcoO of family C are colored in magenta; the 30-mer peptide in family C HCO is colored in yellow. The blue dashed arrows indicate the proton pathways inside each HCO, with the amino acid residues at the entry point of each pathway marked with dashed cycles. (**B**) Structures of Cyt bd-I and bd-II QOs. Protein peptides and heme cofactors are shown as cartoons and sticks with subunits CydA and CydB colored in green and cyan, and CydX in bd-I QOs and CydS in bd-II QOs colored in magenta, respectively. The purple dashed arrow indicates the ‘water-molecule chain’ observed between residues Asp119 (subunit A) and Asp58 (subunit B) in bd-I QOs. The blue dashed arrows indicate two proposed proton pathways in bd-II QOs. Figures are prepared with PyMOL (Molecular Graphics System, LLC) https://www.pymol.org (accessed on 20 December 2021).

HCOs are the most extensively studied terminal oxidases. Despite the variety in the composition of the electron donor, polypeptide, and heme group type, all HCOs possess a conserved redox center composed of a low-spin heme and a heteronuclear heme-copper center (binuclear center, BNC) consisting of a high-spin heme and a copper (Cu_B_) [44,50]. Based on the amino acid sequences and the proton-pumping pathways, members of the HCO superfamily are divided into three families as A, B, and C [51] (Figure 2A).

Bacterial A-family HCOs include *aa*_3_-type C*c*Os (*aa*_3_-HCO, in some cases *caa*_3_-HCO), which exhibit high structural relations to their mitochondrial counterparts, which contain only *a*-type hemes, and the *bo*_3_-type QOs (*bo*_3_-HCO) from *E. coli* (Figure 2A). Most often, A-family HCOs contain three subunits, named SU-I, II, and III. SU-I is highly conserved among all HCOs and typically composed of 12 transmembrane helices (TMHs), which hold the BNC [43]. SU-II contains a membrane-anchored cupredoxin domain functioning for harboring the mixed-valence di-nuclear copper (Cu_A_) acting as the primary electron acceptor [43]. The divalent cations (Mg^2+^ or Mn^2+^) located at the interface between SU-I and SU-II and close to the high spin heme in HCOs of A and C families are not essential for proton pumping; however, their exact functions remain unclear [52,53]. SU-III is present in most bacterial A-family HCOs and possibly influences the oxygen reduction as well as the internal proton flow [43]. An additional subunit (SU-IV) is also identified in A-family HCOs, with its function a mystery yet [10,11,16].

B-family HCOs comprise similar subunit compositions but with low sequence homology to their A-family counterparts (Figure 2A). In contrast to the canonical composition of 12 TMHs in SU-I of A-family HCOs, SU-Is of B-family HCOs, as seen in C*c*Os from *Thermus thermophilus* and *Aquifex aeolicus*, possess 13 and 14 TMHs, respectively [54,55]. The SU-II of B-family HCOs resembles its A-family counterpart in that both contain a membrane-anchored cupredoxin domain; however, an additional subunit (SU-IIa) consisting of a single helix is identified in the former [55]. The His-Tyr cross-link in A-family HCOs, which functions to fix Cu_B_ in a certain configuration and distance from heme *a*_3_ at the BNC [56], is also conserved in B-family HCOs.

C-family HCOs are highly divergent from HCOs of the former two families in protein sequence (Figure 2A). To date, only *cbb*_3_-type C*c*Os are reported in this group [11]. The SU-I of C-family HCOs contains a His-Tyr cross-link as well, but with the two residues residing at two separate helices different from the situations in HCOs of A and B families. In addition, C-family HCOs lack the di-nuclear copper site (Cu_A_) but utilize two auxiliary heme-binding subunits (CcoO and CcoP) to receive electrons from reduced cytochromes [11]. C-family HCO contains an extra subunit CcoQ, a small non-heme protein that is not required for catalytic activity but has a role in the assembly of the HCO complex [57].

The proton pumping in A-family HCOs is performed via two pathways, D-pathway and K-pathway, named accordingly by the conserved and functionally critical residues (Aspartate and Lysine, respectively) near the entry site of each pathway (Figure 2A). In order to pump protons, A-family HCOs utilize the internal ‘proton wires’ to transfer the electronic charges in a way similar to the Grotthuss mechanism [3,58]. Within the longer D-pathway, a series of acid residues and water molecules jointly form a consecutive chain through hydrogen bonds and connect the entrance aspartate to the gating residue close to the BNC [59]. Due to the crucial role of the water molecules, a water gating proton pumping mechanism was thereby proposed in D-pathway [60]. A-family HCOs could be further divided into two types as A1 and A2, based on the residue composition at the hydrophobic end of the D-pathway. Type A1 is featured with a conserved glutamate within the motif XGHPEV on helix VI. However, in type A2, this residue is replaced by consecutive tyrosine and serine in a YSHPXV motif. HCOs of both types A1 and A2 have a covalent bond between one of the Cu_B_-coordinated histidines and a tyrosine on the same helix [61]. The D-pathway is responsible for transporting six protons, four of which are pumped to the positive side (P-side) of the membrane; the remaining two are donated to the active site for use in oxygen reduction [43]. By contrast, the shorter K-pathway typically consists of a few highly conserved polar residues and only qualifies to supply two protons to the catalytic site during the initial reduction of the BNC [59]. A conserved binding domain (carboxyl group) for amphipathic compounds adjacent to the entrance of the K-pathway tends to play a role in organizing the water chain, which supports proton uptake [9,62,63].

The canonical K- and D- pathways are absent in either B-family or C-family HCOs; instead, an alternative K-pathway analogous to that in A-family HCOs is exploited [11,54] (Figure 2A). This K-pathway in B-family HCOs consist of a series of conserved polar residues that form a proton channel. Most of these residues reside within SU-I, with an additional glutamate at the entry site on SU-II. C-family HCOs possess an alternative K-pathway structurally similar to that in B-family HCOs. Within the *cbb*_3_-HCOs from *Pseudomonas stutzeri* and *Rhodobacter sphaeroides*, the proton pathway propagates through a few polar residues with the terminal residue tyrosine cross-linked to one of the histidine ligated to Cu_B_ [11,64].

**Figure 3:**
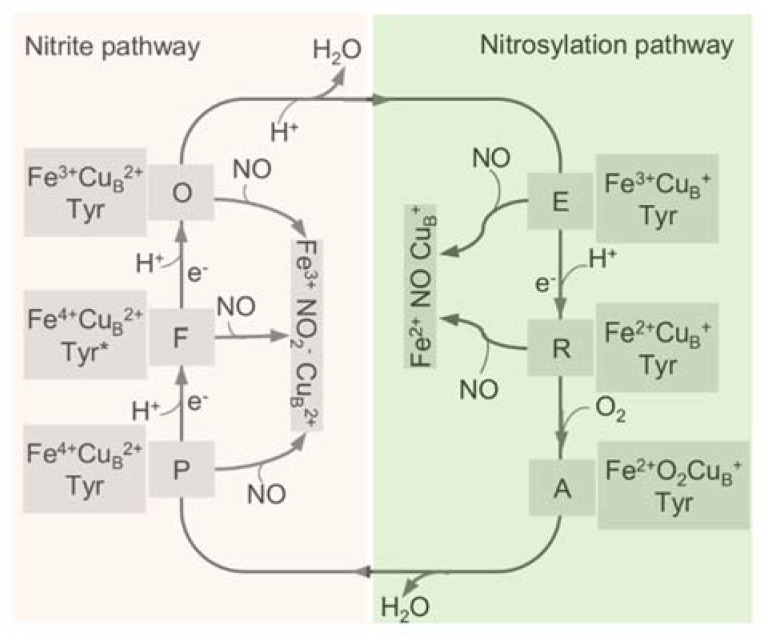
The catalytic cycle of HCO and the interplay with NO through the two pathways. The catalytic cycle of HCO is schematically reported with the indication of the redox and the oxygen ligation state of the BNC (heme a3-CuB active site). In the reductive phase, the oxidized species O is fully reduced to R by two single-electron donations via formation of the half-reduced intermediate E. In the oxidative phase, upon reaction with O_2_, R converts to P and F, and O is regenerated eventually through further electron transfer. The nitrite-bound derivative (Fe^3+^ CuB^2+^ NO_2_^−^) and the nitrosylated adduct (Fe^2+^ CuB^+^ NO) are generated by the reactions of these intermediates with NO. Tyr, CuB-interacting residue Y244, with an asterisk representing the radical form.

All HCOs are electrogenic proton pumps, and the internal and intramolecular electron transfer pathways for each HCO family have been extensively studied [43,65,66,67,68]. The catalytic cycle of A-family HCOs includes two phases, an oxidative phase and a reductive phase, involving several intermediate states of the active site, fully oxidized (**O**, Fe^3+^ Cu_B_^2+^), single-electron reduced (**E**, Fe^3+^ Cu_B_^+^ or Fe^2+^ Cu_B_^2+^), and two-electron reduced (**R**, Fe^2+^ Cu_B_^+^) (Figure 3) [18]. Upon O_2_ binding to **R**, a short-lived new complex **A** is formed, which delivers electrons rapidly to bound O_2_ for the cleavage of the dioxygen bond, forming intermediates **P** and **F** in sequence [69]. Both **P** and **F** are a ferryl derivative of (Fe^4+^ = O Cu_B_^2+^), but the former carries Y244 in the radical form, whereas the latter has Y244 reduced and protonated [31,70,71,72]. Eventually, the fully oxidized state **O** is regenerated from **F** after receiving an additional electron from CuA/heme *a*. During the oxygen reduction, the first proton pumping event occurs during the **P** to **F** transition, and the reaction cycle completes when **R** is regenerated from **E** with two product water molecules released, and two protons pumped [73]. The source of the electron may be a tyrosine or tryptophan radical close to the active site; however, the exact identity of the amino acid, which provides this electron, is still under debate [43].

The proton-pumping loading sites in all HCOs are still controversial [74]. The histidines ligating the heme iron and Cu_B_, as well as the A- and D- propionates of heme *a*_3_ at the catalytic center have been proposed as candidate proton loading sites [75]. A hydrophilic cavity above the hemes, housing divalent cations or water molecules at the interspace of SU-I and SU-II, is possibly the beginning of the water exit pathway [10,11,16].

### 2.2. bd QO

*bd* QO is a quinone-type terminal respiratory enzyme that distributes widely in bacteria and archaea. Unlike HCOs that have two hemes and a copper in the active sites, *bd* QOs accept electrons from quinones (ubiquinol or menaquinol) to reduce oxygen to water using three hemes (Figure 2B) [14,76]. A few isoforms of the *bd* QO family contain only *b*-type hemes, which are less sensitive to inhibition by cyanide [77]. Initially, *bd* QO was considered to consist of two subunits only, named CydA and CydB, encoded by a single operon [78]. However, a small single-transmembrane subunit (CydX or CydS) encoded by the third gene in the *cyd* operon is found to be not only functionally essential but also involved in the assembly of the enzyme complex in some bacteria in recent years [79,80,81]. Based on the structural differences of the quinol binding sites (Q-loop), *bd* QOs are subdivided into L-subfamily (long Q-loop) and S-subfamily (short Q-loop) [14,82].

*bd* QO lacks a counterpart proton-pumping mechanism as in HCOs; instead, they generate a pmf through the transmembrane charge separation and the coupled Q-cycle [83]. There are two potential proton pathways, CydA and CydB pathways, through which protons could pass from the cytoplasm to the high-spin heme site (*b*_595_) in *bd* QO from *Geobacillus thermodenitrificans* [15]. The proton transfer from heme *b*_595_ to the oxygen reduction site via heme *d* is possibly facilitated by water molecules or the heme propionates of heme *b*_595_ [15]. To date, no oxygen channels have been found in *bd* QOs, implying that oxygen molecules likely approach heme *d* laterally from the alkyl chain interface with the membrane [15].

## 3. Roles of HCOs in the Transformation of Nitrogen Oxides

In addition to carrying out O_2_ reduction, HCOs have been shown to be deeply implicated in the biotransformation of multiple nitrogen oxides. It is well recognized that mitochondrial *aa*_3_-HCO is capable of reducing NO_2_^−^ to NO under hypoxic conditions [20,35]. This reduction, which involves only one electron, differs from the four-electron reduction of O_2_ to H_2_O and is not involved with proton pumping [35]. Unlike their eukaryotic counterparts, bacterial HCOs, including the *caa*_3_-HCO and *ba*_3_-HCO from *T. thermophilus*, *cbb*_3_-HCO from *P. stutzeri*, and *bo*_3_-HCO from *E. coli*, are able to catalyze the reduction of NO to N_2_O [36,84,85]. In addition, *cbb*_3_-HCO of *P. stutzeri* is also able to reduce NO_2_^−^ to N_2_O directly [33].

**Figure 4:**
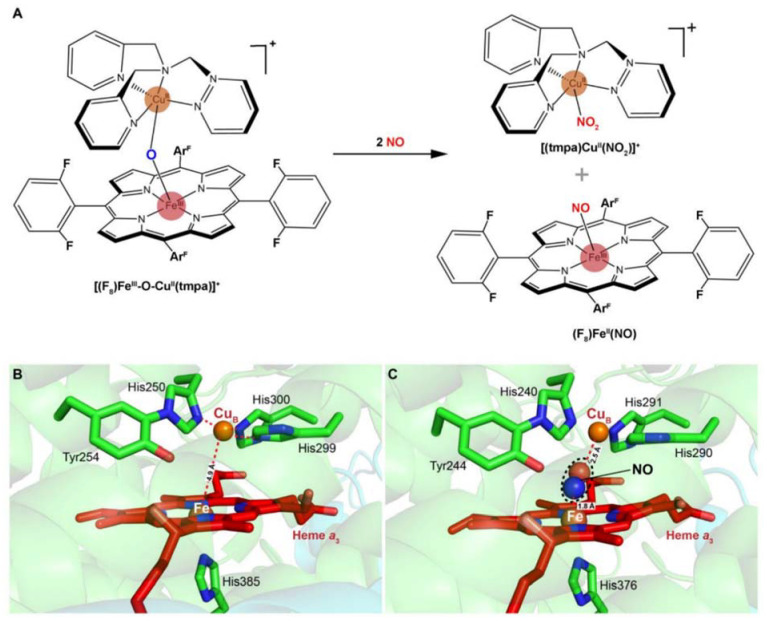
Scheme of a heme-copper assembly mediated oxidation of NO to nitrite and structures of ligand-absent and NO-binding BNCs of CcOs. (**A**) A μ-oxo heme-FeIII-O-CuII complex facilitates NO oxidation to nitrite, forming reduced heme and CuII-nitrito complexes. This scheme is modified from the figure in reference [26] with ChemDraw. (**B**) Spatial structure of the BNC of Cyt caa3 oxidase from *T. thermophilus* HB8. The copper atom (CuB) is coordinated by three histidine residues. The distance between CuB and heme-iron is less than 5 Å. (**C**) X-ray structure of the NO-bound CcO from bovine CcO. The distances between CuB and oxygen atom from NO, heme-Fe, and nitrogen atom from NO are 2.5 Å and 1.8 Å, respectively. Figures of B and C are prepared with PyMOL (Molecular Graphics System, LLC) https://www.pymol.org (accessed on 20 December 2021).

Despite having been known for a long time, the interactions of nitrite with mitochondrial *aa*_3_-HCO have not yet been addressed. Instead, the current understanding of the subject derives mainly from the reactions of nitrite with synthetic heme/copper assemblies [32,86,87] (Figure 4A). During catalytic turnover, the ferrous heme of the *a*_3_-Cu_B_ BNC functions as the electron donor, while the Cu_B_ center serves as a Lewis acid for the cleavage of the N-O bond of nitrite [32]. The overall reaction is a one-electron reduction of nitrite, during which an oxygen atom derived from the nitrite is transferred to the BNC, resulting in an oxo-bridge Fe^3+^-O-Cu^2+^ intermediate. Interestingly, this oxo-bridge Fe^3+^-O-Cu^2+^ could also oxidize NO back to nitrite. It is speculated that the inter-conversion of nitrite and NO has important implications in the modulation of cellular O_2_ balance [32,86,87]. When O_2_ is limited, nitrite interacts with the BNC for reduction, generating NO. This NO molecule can reversibly inhibit the oxygen reduction at the same site, leading to O_2_ accumulation. The reverse reaction is composed of the same steps traversed backward, and as a result, NO is converted back to nitrite for future use, and the enzyme is freed to catalyze the four-electron reduction of O_2_ to water. The overall reaction is regarded as an adaptive mechanism alleviating NO-mediated respiratory inhibition [88].

Multiple lines of evidence suggest that eukaryotic and bacterial HCOs have different reactivities to nitrogen species [89]. In contrast to eukaryotic mitochondrial HCOs, the bacterial counterparts react with nitrite and NO, producing N_2_O as the end product [33,34,89] (Figure 4B,C). Upon the addition of NO to oxidized *ba*_3_-HCO (**O**) of *T. thermophilus*, a six-coordinate heme Fe^2+^-NO species has been detected, suggesting that a hyponitrite (HONNO^−^) ion bound to the BNC in the **E** state (Fe^3+^ Cu_B_^+^) is transiently formed [90]. Further investigations have demonstrated that the binding of two NO molecules to the BNC is accompanied by protonation of the heme *a*_3_-NO species and the electron transfer from Cu_B_^+^, leading to the concomitant formation of the N-N bond. Eventually, N_2_O and H_2_O are released after additional H^+^ is added and the N-O bond is cleaved. This mechanism has been observed in reactions of NO with *caa*_3_-HCO, *bcc*-*aa*_3_ supercomplex (formed by cytochrome *bcc* and *aa*_3_-HCO in *Mycobacterium tuberculosis*), and bacterial NORs, suggesting a possibility that it is conserved in all enzymes of the HCO superfamily [91,92,93,94]. Despite this, the NO reductase activities of all HCOs tested so far are substantially lower than those observed with bacterial NORs, implying that their contribution to NO reduction is likely to be limited unless NORs are absent [85,95]. In addition, the *ba*_3_-HCO of *T. thermophilus* also interacts with nitrite to form a ferrous heme *a*_3_-nitro complex in the BNC, but NO or N_2_O is not produced above the detection limit, suggesting that this enzyme is likely to be susceptible to nitrite inhibition, as discussed below [89].

On the contrary, under reducing conditions, nitrite reacts with *cbb*_3_-HCO to form a six-coordinate ferrous heme *b*_3_-nitrosyl complex [33]. The binding of NO_2_^−^ to heme *b*_3_ triggers electron transfer from the heme to the substrate, leading to its double protonation reduction to NO and release of a H_2_O molecule. Heme *b*_3_ is concomitantly re-reduced with electrons from either Cu_B_ or heme *b*, which is turned toward heme *b*_3_ for creating van der Waals contacts between the hemes [11]. As a result, the ferrous six-coordinate heme *b*_3_–nitrosyl complex is formed, which has NO-trapping activity such that the subsequent catalytic reduction to N_2_O could occur [33]. As the van der Waals contact is missing in other HCO families, it may explain why the reduction of NO_2_^−^ to N_2_O is only observed with *cbb*_3_-HCO [11]. Moreover, given that the reduction of NO by *cbb*_3_-HCO involves a five-coordinate ferrous heme *b*_3_–nitrosyl adduct [56], five- and six-coordinate heme *b*_3_–nitrosyls likely exist in equilibrium in the reduction, and their ratio may vary depending on experimental conditions [33].

## 4. Inhibition of HCOs by Nitrite and NO

To date, a large number of HCO inhibitors have been known, and the repertoire is still expanding [96,97]. These HCO inhibitors interact with the BNC directly and thus are substantially less effective on other terminal oxidases that do not pump protons. In general, HCO inhibitors can be divided into four categories: (i) heme-binding inhibitors competitive with oxygen (CO and NO, etc.); (ii) heme-binding inhibitors not competitive with oxygen (cyanide (CN^−^), H_2_S, and azide (N_3_^−^), etc.); (iii) inhibitors which act by preventing binding of cytochrome *c* to C*c*O (alkaline proteins and polypeptides, etc.); (iv) Quinol-like compounds acting at the Q binding site of QOs [97].

Inhibition of the BNC by CN^−^, H_2_S, and N_3_^−^ has been intensively investigated, offering the best illustration of the mechanisms underpinning the oxygen-binding and proton pumping [98,99]. Three forms of mitochondrial *aa*_3_-HCOs in the **O** state have been defined by the difference in the cyanide binding rates, namely, “slow”, “fast”, and “open” forms [100,101]. During the catalytic turnover, the open form resulting from O_2_ oxidation of the **R** state has Fe^3+^-OH^−^ as the iron coordination structure of the O_2_ reduction site, allowing proton pumping after each of the first and second single-electron donations to the fully oxidized enzyme [102]. The nature of the ligands that bind to the BNC is still uncertain, and the proposed include a peroxide group bridging the two metal sites in the O_2_ reduction site (Fe^3+^-O-O-Cu^2+^), superoxide, hydroxide, water, and even oxygen [103,104,105]. Most recently, it has been suggested that a radical Tyr-288 is present in the fast form and a protonated Tyr-288 in the slow form [106]. Nonetheless, in contrast to the open form, neither the fast form nor the slow form induces proton pumping [102]. As the rate of CN^−^ binding to the open form is substantially higher than the binding rate to the other two forms; the proton-pumping is highly sensitive to cyanide [107].

The reactions between mitochondrial *aa*_3_-HCO and NO were first described more than four decades ago [29]. The NO inhibition of *aa*_3_-HCO is rapid and reversible and may occur in competition with oxygen. Inhibition takes place following two different pathways, the nitrosylation pathway and the ‘nitrite’ pathway [30] (Figure 3). In the nitrosylation pathway, a relatively stable (Fe^2+^ NO Cu_B_^+^) nitrosyl-derivative of the **R** state is formed when **E** or **R** reacts with NO [31,71,88,108]. Given that the stable nitrosyl adduct can be formed at a rate comparable to that of O_2_, it is apparent that the reduction of the BNC and its stabilization in the nitrosylated state are equally favored [109]. One of the peculiar properties of the stable nitrosyl adduct is that it would not modify NO, and as a result, NO can be released unaltered [31]. This likely explains why mitochondrial *aa*_3_-HCO loses the ability to reduce NO to N_2_O because NO can be released [110]. In addition, the rate of NO dissociation from reduced *aa*_3_-HCO is unusually high, at least an order higher than that of other hemoproteins such as hemoglobin [111]. A consequence is that the activity can be rapidly recovered after NO is removed, although *aa*_3_-HCO is promptly inhibited upon NO exposure.

Alternatively, upon reacting with HCO, NO can be transformed by the BNC of other intermediates to form the nitrite-bound enzyme [71,88]. In this ‘nitrite’ pathway, NO is oxidized to nitrite by the **O** BNC in either slow or fast form, by **P**, and by **F** [29,31,70,71,112,113] (Figure 3). When encountering the fast HCO, NO initially interacts with the oxidized Cu_B_ rather than heme *a*_3_, forming a Cu_B_^+^-NO^+^ complex, which is subsequently hydroxylated to generate nitrite, a proton, and Cu_B_^+^ [108]. During the reaction, one electron is then made available at the BNC, allowing its re-equilibrating rapidly with heme *a* via reverse electron transfer. In the end, the heme *a*_3_ nitrosyl complex is formed, and the enzyme is inhibited [108,114]. Inhibition of HCOs by NO through these ‘nitrite’ pathways, similar to the nitrosylation pathway, is reversible, and activity is restored by expelling nitrite from the heme *a*_3_ nitrosyl complex [71,115].

Consistent with the independent discovery of these two pathways, they coexist upon NO exposure [29,40]. One pathway that prevails over the other depends on the turnover conditions and concentration of NO and physiological substrates, cytochrome *c* and O_2_ [111]. When O_2_ and NO are allowed to react at the same time with the enzyme, inhibition by NO may or may not occur in competition with O_2_, depending on the fractional distribution of the catalytic intermediates of *aa*_3_-HCO. Since Oxygen can only bind to the **R** BNC [116], which is the only intermediate that can react with both O_2_ and NO, and the BNC in the states **O**, **E**, **P**, and **F** can solely react with NO, the reaction of NO with these latter intermediates does not occur in competition with O_2_ [30,117,118,119].

Both pathways have been suggested to contribute to the resistance of *bd* QO to NO, although the molecular mechanism at the basis of inhibition of this enzyme by NO has not been fully elucidated [120]. Cu_B_ within HCOs plays an important role in determining the reaction rate with NO, and the NO dissociation rate from the nitrosyl adduct formed [41]. In the Cu-lacking *bd* QO, heme *b*-595 mimics the role of Cu_B_ in HCOs to react with NO at a rate that is remarkably slow compared to the HCO BNC, limiting nitrite production (via the ‘nitrite’ pathway) [114,120,121,122]. Moreover, although the *bd* enzyme also interacts with NO to form the nitrosyl adduct through the nitrosylation pathway, it has an NO dissociation rate substantially faster than that of HCOs [42,120,123]. The property results in prompt restoration of the enzyme activity with decreasing concentrations of NO. Interestingly, the mycobacterial *bcc*-*aa*_3_ supercomplex is hyperresistant to NO inhibition (at 30 μM) more than other HCOs and even *bd* QO, which exhibit reduced activity in the presence of NO in the nanomolar range [42]. It is proposed that upon NO exposure, the newly formed nitrite by this *bcc*-*aa*_3_ supercomplex may not bind to the heme *a*_3_ moiety, presumably due to low affinity, and as a consequence, is expelled immediately from the enzyme without compromising oxygen reduction [94].

Similar to NO, nitrite also displays inhibitory effects on HCOs [22]. As nitrite can be transformed by a variety of proteins, including HCO, into other nitrogen oxides, especially NO, its inhibition has been attributed to NO for decades [124]. Thus, to date, investigations into the mechanisms underpinning the inhibition of HCOs by nitrite have been scarce. Nevertheless, in vitro biochemical analyses have demonstrated that the inhibitions of hemoproteins by the two molecules are not identical, albeit similar [125,126]. Although both NO and nitrite involve the formation of a ferrous-nitrosyl (Fe^2+^-NO) complex upon binding to the ferrous ion within heme, leading to dissociation of the proximal histidine ligand [126], NO interacts with heme to directly form the Fe^2+^-NO complex, whereas it is a two-step process for nitrite as nitrite has to be converted to NO first [20,36,127]. In recent years, new chemistries between nitrite and hemoproteins have been revealed [128]. Upon interaction of nitrite with hemoproteins, the iron-nitrosyl product is formed and subsequently enters a nitrite reductase/anhydrase redox pathway converting two molecules of nitrite into dinitrogen trioxide (N_2_O_3_) [129]. N_2_O_3_ may then nitrosate proteins and reconstitute NO via homolysis to NO and NO_2_^•^. Meanwhile, nitrite is found to increase H_2_O_2_ levels by directly inhibiting catalase or reacting with hemoproteins under O_2_ replete conditions to generate H_2_O_2_, introducing oxidative stress on cells [128,130]. Clearly, further studies are required to determine the contribution of the secondary effects induced by nitrite, including the N_2_O_3_ formation and the oxidative stress, to the inhibition of nitrite.

## 5. HCOs Are Primary Targets of Nitrite but Not NO In Vivo

Upon exposure to nitrite, the most evident phenotype of bacterial cells is impaired growth, which underlies the long history of using the nitrogen oxide as a preservative in meat products [131]. From earlier studies of growth inhibition by nitrite, two hypotheses have been proposed to explain the nature of the inhibition. In *Pseudomonas aeruginosa*, nitrite was initially found to inhibit active transport, oxygen uptake, and oxidative phosphorylation, thereby reducing aerobic respiration [39]. Later, it was demonstrated by in vitro analyses that nitrite directly compromises *cbb*_3_-HCO of *P. aeruginosa* [39,132]. Such a phenomenon was not observed with bacteria without an HCO, such as *Clostridium* species, in which early investigations into the antimicrobial mechanism of nitrite were mostly conducted as the toxin produced from *Clostridium botulinum* is of particular concern in vacuum-packaged meat products [39]. Instead, it had been suggested in *Clostridium* species that the growth defect caused by nitrite is linked to pyruvate-ferredoxin oxidoreductase that carries a single Fe-S cluster, whose inactivation results in the accumulation of pyruvate [133,134]. Despite the lack of direct evidence, the inhibition of this enzyme by nitrite is proposed to be carried out by reaction of nitrite-derived NO rather than by nitrite per se [134]. The idea that nitrite inhibition depends on NO was boosted by the finding reported in 1983 that iron-sulfur proteins in vegetative cells of *C. botulinum* react with added nitrite to form iron-NO complexes, with resultant destruction of the iron-sulfur cluster [124]. Ever since, it has been widely considered that the antibacterial effects of nitrite are attributable to NO formation.

**Figure 5:**
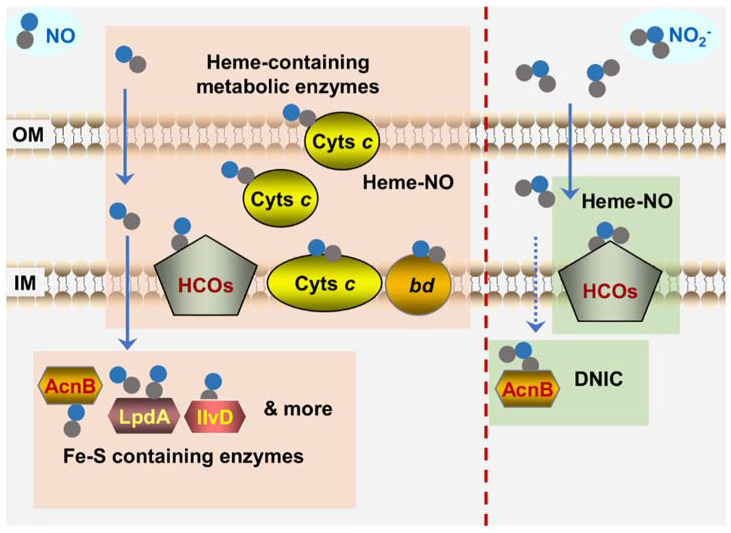
Bacterial targets of NO and nitrite revealed by in vivo analyses. Shown is the scenario that bacteria are confronted with NO from exogenous sources. The proteins sensitive to NO primarily include: (i) Fe-S containing dehydratases such as LpdA, IIvD, and AcnB that can form DNIC with NO; (ii) hemoproteins such as cyts c, HCOs, and cyt bd quinol oxidase that can form Fe^2+^-NO complex. Unlike NO, the targets of nitrite are more specific. HCOs are the most crucial targets of nitrite. Besides, housekeeping aconitase AcnB rapidly loses activity upon nitrite exposure. Dashline arrow for the transport of nitrite represents that the molecules, unlike NO, could not diffuse into the cytoplasm easily. OM and IM represent outer- and inner-membrane of Gram-negative bacteria, respectively. Solid and dash line arrows represent crossing the membranes freely and in a transporter-dependent manner respectively.

Given that NO attracts all the attention, attempts aiming at identifying bacterial proteins susceptible to NO have been repeatedly made, especially with high-throughput screening approaches. Not surprisingly, a broad array of redox-active enzymes have been identified to be susceptible to NO, including aconitase, argininosuccinate synthase, dihydroxyacid dehydratase, fructose-1,6-biphosphate aldolase, pyruvate dehydrogenase, lipoamide dehydrogenase, and α-ketoglutarate dehydrogenase, most of which carry a Fe-S cluster (Figure 5) [28,135,136,137,138,139]. Importantly, NO targets associated with bacteriostasis differ significantly from one species to another, in some cases even within the same species, implying that the homeostasis of redox-sensitive proteins may largely underpin the substantial difference in NO-targets identified to date among different bacteria [138]. Unexpectedly, although HCOs and *bd* QOs have been individually identified to be NO-targets [42,123,140,141,142], they, even hemoproteins, were not among the identified in two comprehensive screenings of *E. coli* and *Salmonella enterica* serovar Typhimurium (*S.* Typhi), suggesting that they do not belong to the primary NO-target repertoire linked to the NO-caused growth arrest [137,138].

However, a twist took place recently where cyts *c* have been identified to be crucial targets of NO in bacteria that are particularly rich in this type of protein, such as *Shewanella oneidensis*, which is renowned for respiratory versatility largely owing to 42 different cyts *c* [143,144,145] (Figure 5). In vitro biochemical analyses have firmly established that cyts *c*, the same as other hemoproteins, are highly susceptible to both NO and nitrite, although they differ from them in that they contain one or several heme molecules through covalent linkages [36,146]. More importantly, as all cyts *c* are located exclusively outside the cytoplasm (either membrane-bound or soluble in the periplasm of Gram-negative bacteria), they are at the forefront of the attack by exogenous NO and nitrite. In *S. oneidensis*, the overall cyt *c* content rather than any individual cyt *c* dictates the susceptibility to NO because the loss of all cyts *c*, but not any single one of them, elicits a drastic difference in the sensitivity of the cells to NO (wild-type vs. cyt *c*-deficient strains) [147]. Moreover, the NO tolerance of *S. oneidensis* increases with the overall cyt *c* abundance that is manipulated by up-regulation of cyt *c* biosynthesis [147]. A similar scenario has been observed in *E. coli*, despite its relatively low cyt *c* abundance, which confers the cell′s NO tolerance less effectively.

In cyt *c*-deficient mutants of *S. oneidensis*, metabolic enzymes were identified to be the NO targets in other bacteria that are critically linked to the impairment caused by NO growth, such as aconitase and dihydroxyacid dehydratase, become hypersensitive to NO [147] (Figure 5). Based on all of these findings, it has been proposed that cyts *c* appear to function as a major NO sink, especially in bacteria rich in such proteins, resulting in reduced intracellular levels of free NO and thus protecting other growth-critical targets. Since NO dissociation from the ferrous-nitrosyl (Fe^2+^-NO) complexes is the rate-limiting process [36], this sinking effect is conceivably not likely to be very transient. Furthermore, this proposal offers one possible explanation for the failure that cyts *c* are not among the NO targets identified in *E. coli* and *S. Typhi* because they are not involved in aerobic growth, in which QOs (non-cyt *c* HCOs) are employed to respire oxygen [44].

In contrast to NO, HCOs are the specific and primary cellular target of nitrite. *S. oneidensis* and *E. coli* strains devoid of *bd* QO are highly sensitive to nitrite, whereas the loss of HCO (*cbb*_3_-HCO and *bo*-HCO for *S. oneidensis* and *E. coli*, respectively) does not affect nitrite tolerance [148,149,150] (Figure 5). On the contrary, *bd* QO is dispensable to NO inhibition. When the cyt *c* contents are produced at similar levels, the presence of *bd* QO or not does not introduce a significant impact on growth [80,147,148,151]. These findings are in line with the properties of HCOs and *bd* QO revealed in in vitro analyses that the former are substantially more sensitive to nitrite than the latter in bacteria [42,152]. Moreover, the influence of cyts *c* on nitrite-induced bacteriostasis is negligible as the depletion of all cyts *c* only affects the susceptibility to NO but not to nitrite [147].

Further investigations into the NO-independent impacts of nitrite on HCOs have demonstrated that the essence of nitrite inhibition is proton translocation [153,154,155,156]. In *P. aeruginosa*, nitrite could not only prevent biofilm formation on the apical surface of airway epithelial cells but also modulate susceptibility to aminoglycosides by inhibiting bacterial respiration and oxygen uptake [157,158,159]. While HCOs and *bd* QOs differ from each other substantially in proton translocation efficiency, they appear similar in oxygen consumption [5,14,156]. A systematic survey of all types of HCOs with respect to their impacts on aminoglycoside susceptibility, including *aa*_3_-HCO from *Bacillus subtilis* and *Staphylococcus aureus*, *caa*_3_-HCO from *S. oneidensis*, *cbb*_3_-HCO from *S. oneidensis* and *P. aeruginosa*, and *bo*_3_-HCO from *E. coli*, have demonstrated that nitrite confers the cell’s increased tolerance to a variety of aminoglycosides by inhibiting all of these HCOs under test [148,156]. This effect coincides with that caused by pmf destroyers CCCP and KCN, but is in contrast to that resulting from bedaquiline, which affects respiration (oxygen consumption) but not pmf. Thus, the mechanism underlying the modulation of aminoglycoside susceptibility by nitrite is to inhibit HCOs by abolishing proton translocation via proton pumping [153,156].

In addition to the NO-independent inhibition of HCOs by nitrite, recent advances have revealed an array of physiological aspects in which nitrite and NO function independently from each other. These include enzymes for their formation and detoxification and novel biological activities such as the lowing effect of nitrite on blood pressure [22,160]. In addition, nitrite and NO as signal molecules at very low concentrations are perceived by completely different sensing systems, through which they are linked to different biological processes [22,161]. Moreover, in recent years, nitrite has been gradually rediscovered as a beneficial molecule, either endogenously formed or therapeutically added [162,163]. Nitrite (nebulized sodium nitrite) is currently in a phase 2b clinical trial as a drug for pulmonary hypertension treatment on the basis of the finding that it could be safely applied to reach millimolar concentrations in the airway surface liquid [164]. These findings from in vivo studies would undoubtedly, in turn, prompt more focused investigations into the mechanistic differences in the interactions of proteins to NO and nitrite.

## 6. Concluding Remarks

HCOs are transmembrane enzymes that catalyze the reduction of O_2_ to H_2_O, through which the energy is conserved as a proton gradient across the membrane via electrogenic chemistry and proton pumping. As redox-sensitive hemoproteins, HCOs readily interact with nitrogen oxides, nitrite, and NO in particular. While the interaction enables these enzymes to carry out biotransformation of nitrogen oxides, it also results in inhibition of catalytic activity. HCOs of eukaryotes are capable of reducing NO_2_^−^ to NO, but most of their prokaryotic counterparts reduce NO to N_2_O. Nevertheless, this reduction is not associated with proton pumping as it involves only one electron. Inhibition of HCOs by NO is rapid and reversible, which occurs in competition with oxygen only in the fully reduced state. In contrast, HCOs in the partially reduced and fully oxidized form can solely react with NO. Despite the lack of direct evidence, the inhibition of HCOs by nitrite is proposed to be via NO.

The importance of nitrite in the physiology of living organisms has gone unrecognized since the introduction of the notion that nitrite has effects through the formation of NO. Despite this, it is now clear that nitrite and NO are largely seen as two distinct nitrogen oxides by bacterial cells, although they share some common biochemical properties. NO appears to interact with most, if not all, redox-sensitive proteins, whereas nitrite shows high specificity. In particular, NO indiscriminately interacts with a variety of cytochromes *c*, presumably all hemoproteins, but nitrite inhibits HCOs specifically. Naturally, there must be mechanistic differences in the inactivation of HCOs by nitrite and NO. It should be noted that although bacterial HCOs share the common core architecture with more complex eukaryotic counterparts, they evolve through long-term adaptation, varying environments to possess distinctive features that allow them to exploit a much broader set of chemistries. However, compared to eukaryotic HCOs, structural and mechanistic studies to elucidate biochemical principles underlying the interaction between bacterial HCOs and nitrogen oxides remain limited, and are urgently needed.

In recent years, diverse and different solutions to respond and cope with nitrite and NO in bacteria have been revealed, relying on the sensory proteins that are extremely sensitive to changes in the concentrations of the chemicals. One may imagine that these sensors, mainly hemoproteins, can be exploited to address the mechanistic differences in interacting with nitrite and NO. More excitingly, in addition to NO, which has been administrated in clinical settings for decades, nitrite is now on the journey from toxin to therapy. It is, therefore, optimistic to predict that novel and surprising mechanisms, which reconcile the similar biochemical properties with the different physiological impacts of NO and nitrite, will be unraveled in living organisms, especially microorganisms, because they do not conform to boundaries.
